# Novel *Synechococcus* Genomes Reconstructed from Freshwater Reservoirs

**DOI:** 10.3389/fmicb.2017.01151

**Published:** 2017-06-21

**Authors:** Pedro J. Cabello-Yeves, Jose M. Haro-Moreno, Ana-Belen Martin-Cuadrado, Rohit Ghai, Antonio Picazo, Antonio Camacho, Francisco Rodriguez-Valera

**Affiliations:** ^1^Evolutionary Genomics Group, Departamento de Producción Vegetal y Microbiología, Universidad Miguel HernándezSan Juan de Alicante, Spain; ^2^Institute of Hydrobiology, Department of Aquatic Microbial Ecology, Biology Center of the Academy of Sciences of the Czech RepublicČeské Budějovice, Czechia; ^3^Cavanilles Institute of Biodiversity and Evolutionary Biology, University of ValenciaValencia, Spain

**Keywords:** *Synechococcus*, picocyanobacteria, freshwater reservoirs, metagenomics, abundance, smallest estimated size

## Abstract

Freshwater picocyanobacteria including *Synechococcus* remain poorly studied at the genomic level, compared to their marine representatives. Here, using a metagenomic assembly approach we discovered two novel *Synechococcus* sp. genomes from two freshwater reservoirs Tous and Lake Lanier, both sharing 96% average nucleotide identity and displaying high abundance levels in these two lakes located at similar altitudes and temperate latitudes. These new genomes have the smallest estimated size (2.2 Mb) and average intergenic spacer length (20 bp) of any previously sequenced freshwater *Synechococcus*, which may contribute to their success in oligotrophic freshwater systems. Fluorescent *in situ* hybridization confirmed that *Synechococcus* sp. Tous comprises small cells (0.987 ± 0.139 μm length, 0.723 ± 0.119 μm width) that amount to 90% of the picocyanobacteria in Tous. They appear together in a phylogenomic tree with *Synechococcus* sp. RCC307 strain, the main representative of sub-cluster 5.3 that has itself one of the smallest marine *Synechococcus* genomes. We detected a type II phycobilisome (PBS) gene cluster in both genomes, which suggests that they belong to a phycoerythrin-rich pink low-light ecotype. The decrease of acidic proteins and the higher content of basic transporters and membrane proteins in the novel *Synechococcus* genomes, compared to marine representatives, support their freshwater specialization. A sulfate Cys transporter which is absent in marine but has been identified in many freshwater cyanobacteria was also detected in *Synechococcus* sp. Tous. The RuBisCo subunits from this microbe are phylogenetically close to the freshwater amoeba *Paulinella chromatophora* symbiont, hinting to a freshwater origin of the carboxysome operon of this protist. The novel genomes enlarge the known diversity of freshwater *Synechococcus* and improve the overall knowledge of the relationships among members of this genus at large.

## Introduction

Cyanobacteria are among the most diverse and widely distributed group of bacteria. They are the only prokaryotes capable of performing oxygenic photosynthesis, and greatly contribute to global primary production, fixing a substantial amount of carbon both in marine and freshwater environments. Among them, unicellular picocyanobacteria (Pcy) are globally distributed in almost all lakes and oceans. Typically, Pcy clades (*Synechococcus, Prochlorococcus*, and *Cyanobium*) are designated as non-bloomers (Steffen et al., 2012) and are either single or colonial rod-shaped cells ranging from 0.2 to 3 μm. However, they can form dense populations of up to several million cells per ml at the deep chlorophyll maximum (DCM) of mesotrophic stratified lakes (Camacho et al., 2003a,b) and, though less abundant, they also peak at the DCM of oceans and some seas (Partensky et al., 1999). The genera *Synechococcus* and *Cyanobium* are the dominant picocyanobacteria in freshwater systems (Callieri, 2008). However, the number of strains of marine origin with their genomes sequenced is much larger than their freshwater counterparts, providing a rather incomplete picture of the diversity of the genus. Furthermore, freshwater *Synechococcus* strains play a critical role in the ecological health of water bodies that are important human resources.

Similar to other cyanobacteria, *Synechococcus* ecotypes display differences in their accessory pigments and phycobilisomes (PBS) that make them adapted to different wavelengths of light (Camacho et al., 2000). *Synechococcus* PBS, responsible for light absorption and energy transfer to chlorophyll a (photosystem II) for the photosynthesis process, are also diagnostic and important for the type of light range spectrum in which they live, providing an advantage for some *Synechococcus* ecotypes in deep ecosystems with low light (Scanlan et al., 2009). There are three major types within this genus depending on the phycobiliprotein genes that they contain: Type I strains contain only C-phycocyanin resulting in green pigmentation; type II strains contain C-phycocyanin and phycoerythrin I and produce pink pigmentation; type III strains contain phycocyanin, phycoerythrin I and phycoerythrin II, presenting a wide range of pigmentation and some of them display chromatic adaptation (Six et al., 2007; Dufresne et al., 2008). Moreover, the morphometry and trophic state of lakes and ponds strongly influences composition, diversity and abundance of Pcy communities (Callieri, 2008; Callieri et al., 2012). Typically, deep, clear and oligotrophic/mesotrophic lakes contain mainly phycoerythrin (PE) rich cells while in shallow, turbid (humic) eutrophic lakes phycocyanin (PC) rich cells predominate (Callieri and Stockner, 2002). The success of *Synechococcus* in oligotrophic systems is explained by its capacity for adaptation to low-light conditions (Callieri et al., 2012). Their affinity to orthophosphate and other organic phosphorous sources apart from inorganic phosphates and their capacity for nitrogen storage in phycobilins (Camacho, 2006) enhance *Synechococcus* competition against algae and other bacteria (Vadstein, 2000).

Analysis of 16S rRNA genes and the internal transcribed spacer (ITS) of *Synechococcus* clearly suggests a polyphyletic nature (Robertson et al., 2001). These studies have revealed the existence of three marine sub-clusters: 5.1, 5.2, and 5.3 (Fuller et al., 2003; Scanlan et al., 2009; Mazard et al., 2012), and 13 clusters of non-marine Pcy (Callieri et al., 2013). Cluster 5.1 encompasses most marine clades (Rocap et al., 2003; Dufresne et al., 2008) but the less studied clusters 5.2 and 5.3 appear to be very important to understand the evolution of *Synechococcus* and *Cyanobium* and have unexpected relations to non-marine strains (Callieri et al., 2013). Recently, a new group, ‘halotolerants’ from a Mexican athalassohaline crater-lake has been found to be very close to the marine subcluster 5.3 (*Synechococcus* sp. RCC307), demonstrating that euryhaline and marine strains affiliate closely (Callieri et al., 2013). Phylogeny and ancestral state reconstruction approaches have shown that the earliest Pcy lineages were freshwater inhabitants, whose communities possess greater diversity than marine Pcy (Sanchez-Baracaldo et al., 2005, 2008; Blank and Sanchez-Baracaldo, 2010).

Although a large number (33) of marine *Synechococcus* strains have been sequenced (Scanlan et al., 2009) only a few (6) freshwater genomes are available. Among them, *Synechococcus elongatus* (Frenkel et al., 1950) plays now an important role as a model organism used in genetic engineering. Additional genomes, e.g., *Synechococcus* sp. GFB01 from a Brazilian Amazon lagoon (Guimarães et al., 2015), strains JA-3-3Ab and JA-2-3B’a(2-13) from Yellowstone (Bhaya et al., 2007) and PCC6312, PCC7502 freshwater representatives (Shih et al., 2013) were sequenced in the last few years. Metagenomic studies have suggested that Pcy (including *Synechococcus*) are far outcompeted by filamentous cyanobacteria (Ghai et al., 2012) in hypertrophic systems (e.g., Albufera de Valencia, Spain) but can be found in significant numbers in mesotrophic reservoirs (Oh et al., 2011; Ghai et al., 2012). Remarkably, it has been suggested that only a single abundant species of *Synechococcus* occurs in Lake Lanier (located in Georgia, United States), as all overlapping metagenomic reads belonging to *Synechococcus* were restricted to nearly 98–99% average nucleotide identity (ANI) (Oh et al., 2011).

In this manuscript we have used genome reconstruction from metagenomics (Ghai et al., 2014) to identify the most abundant *Synechococcus* genome obtained from a metagenomic sample of the freshwater reservoir of Tous (Valencia, Spain), an oligotrophic to slightly mesotrophic environment with high abundance of *Synechococcus*. We also assembled a highly similar *Synechococcus* relative from a Lake Lanier metagenome (Oh et al., 2011) through the same approach. This metagenomics genome reconstruction approach has provided a global view of the genomic properties and ecophysiological features of these two microbes. They represent an important and hitherto unknown species of freshwater *Synechococcus* with a widespread distribution.

## Materials and Methods

### Sampling Site Description and Physicochemical Profiles

The Tous New Dam is located in the lower course of the river Júcar, in the province of Valencia (Eastern Spain). A total catchment area of 17,821 km^2^ leads to the Tous dam, which is the last and one of the largest of a system of eight regulating reservoirs along the Júcar (Xúquer) river. It is an embankment-type dam with a clay core that has been rebuilt on top of an older dam that suffered severe failure in 1982 during a major flood. It has a standard maximum water surface elevation of 32.5 m corresponding to 340 × 106 m^3^ total water capacity, occupying an area of 9.80 km^2^, although its flood-regulating capacity reaches 700 × 106 m^3^.

Detailed vertical profiles of physical and chemical parameters were obtained with a CTD probe (SeaBird 19) equipped with specific sensors for pressure, conductivity and temperature. The probe was also fitted with sensors for oxygen (SB-43), chlorophyll-a (fluorimetric, Wetstar), phycoerythrin (fluorimetric, Seapoint), phycocyanin (fluorimetric, Cyclops-7 Turner), turbidity (Seapoint 880 nm) and c-DOM (WETStar Ex370/Em460). Sampling was performed on February 20th, 2015 when the reservoir was thermally mixed. Sampling depths were chosen on the basis of the *in situ* acquired physical and chemical profiles (Supplementary Figure S1). The samples were obtained from a boat, moored ∼500 m from the dam near the deepest point of the reservoir, by using a double cone fine layer sampler (Camacho, 2006). Water samples for chemical analyses were obtained from the same sample used for metagenomics analysis and a surface sample was also analyzed. These samples were filtered (e.g., for dissolved nutrient analyses) through Whatman GF/F filters, then poured into glass or PVC bottles, both previously washed with acid, and subsequently preserved at 4°C until analysis.

Major chemical variables including inorganic soluble forms of nitrogen (nitrate and ammonium) and phosphorus (soluble reactive phosphorus, SRP), as well as total nitrogen (TN) and total phosphorus (TP), were determined according to Standard Methods (American Public Health Association et al., 1915). Total organic carbon (TOC) was determined on a Shimadzu TOC-VCSN Analyser. Photosynthetic pigments were obtained by HPLC after extraction in acetone (Picazo et al., 2013).

### Sampling, Sequencing, and Annotation

Two samples were taken for metagenomic analyses from the Tous reservoir on February 20th, 2015 at 12 and 25 m depths. Approximately 40 L of water from each sample was sequentially filtered through series of 20, 5, and a 0.22 μm-pore-size polycarbonate filters (Millipore). DNA extraction was only performed on the 0.22 μm filter for both depths as previously described (Ghai et al., 2010). Briefly, filters were treated with final concentrations of 1 mg/mL lysozyme and 0.2 mg/mL proteinase K and DNA was extracted with the Power isolation soil kit (Mo Bio). Summary statistics of sequencing was performed using Illumina HiSeq 4000 (Macrogen, South Korea), with an insert size of 350 bp, obtaining an average read length of 79.4 bp. A total of 284 and 281 million sequence reads (PE 2x100 bp) representing 28 and 27 Gb of sequence data were produced for 12 and 25 m 0.22 μm fraction, respectively. Each data set was assembled independently using the IDBA-UD assembler (Peng et al., 2012) with the following parameters: mink 70, maxk 100, step 10, pre-correction. Gene predictions on the assembled contigs were done using Prodigal in metagenomic mode (Hyatt et al., 2010), tRNAs were predicted using tRNAscan-SE (Lowe and Eddy, 1997) and ribosomal rRNA genes were identified using ssu-align (Nawrocki, 2009; Nawrocki and Eddy, 2010) and meta-rna (Huang et al., 2009). Comparisons of predicted protein sequences against NCBI NR, COG (Tatusov et al., 2001) and TIGFRAM (Haft et al., 2001) databases were performed for taxonomic binning and functional annotation. The same assembly and annotation procedure was used for contigs from the Lake Lanier (Oh et al., 2011). The samples used to reconstruct the Lanier genome were Lanier S2 (August 28, 2009) and Lanier S3 (September 7, 2009) both generated by Illumina GAII instrument. Summary statistics of the Tous and Lanier samples used for *Synechococcus* genome reconstruction is provided in Supplementary Table S1. Previously described assembled fosmid contigs (>10 Kb) from Amadorio were also annotated similarly and 151 cyanobacterial fosmids were selected (Ghai et al., 2014).

### 16S rRNA Reads Classification

A non-redundant version of the RDP database was prepared by clustering all available 16S rRNA coding sequences (approximately 2.3 million) into approximately 800,000 sequences at 90% identity level using UCLUST (Edgar, 2010). This database was used to identify candidate 16S rRNA fragments among the Illumina reads (unassembled). If a sequence matched this database at an *e*-value < 1e-5 it was considered a potential 16S rRNA fragment. These candidate fragments were aligned to archaeal, bacterial and eukaryal 16S/18S rRNA HMM models using ssu-align to identify true 16S/18S sequences (Nawrocki, 2009). The 16S rRNA fragments retrieved were compared to the entire RDP database and classified into a high level taxon if the sequence identity was ≥80% (BLASTN) and the alignment length was ≥90 bp. Fragments failing these thresholds were discarded.

### Identification of Cyanobacterial Contigs and Genome Reconstruction

Contigs longer than 5 Kb were used for the *Synechococcus* sp. Tous and *Synechococcus* sp. Lanier genome reconstructions. Contigs were considered cyanobacterial if >60% of genes gave top BLAST hit to Cyanobacteria. More specifically, contigs with >60% of genes with top BLAST hit to *Synechococcus* sp. RCC307 were grouped using taxonomy, principal component analysis of tetranucleotide frequencies, GC content and coverage values in Tous and Lanier metagenomes (Oh et al., 2011). Tetranucleotide frequencies were computed using wordfreq program in the EMBOSS package (Rice et al., 2000). Principal component analysis was performed using the FACTOMINER package in R (Lê et al., 2008).

To improve the completeness and remove redundant contigs of the novel freshwater *Synechococcus* sp. Tous assembled genome, a second assembly step was performed. Tous metagenome reads were mapped using BWA (Li and Durbin, 2009) to *Synechococcus* contigs (>5 Kb contigs from 12 to 25 m metagenomes of Tous) and an additional 151 cyanobacterial contigs (>10 Kb) obtained from a fosmid library made from the metagenome of the Amadorio reservoir (Ghai et al., 2014). The mapped reads were reassembled along with the contigs using SPAdes (Bankevich et al., 2012). Finally, a composite *Synechococcus* genome comprising multiple highly related clones was extracted by considering contigs that grouped together based on GC%, PCA, and abundances in the Tous datasets. Similarly, for *Synechococcus* sp. Lanier we used *Synechococcus* contigs from two Lake Lanier metagenomes SRA029314.1 (S2), SRA029315.1 (S3) (Oh et al., 2011) for the reassembly and binning step to obtain the final genome. To estimate the genome size and completeness of the assembled genomes two different universal gene sets were used, one with 35 genes (Raes et al., 2007) and another with 111 genes (Albertsen et al., 2013).

### Phylogenomic Tree

A total of 35 representative genomes from marine, euryhaline, and freshwater *Synechococcus* for each clade and sub-cluster (Scanlan et al., 2009; Shih et al., 2013) were used together with the two novel *Synechococcus* in a reference protein-concatenate-based phylogenomic tree. Three complete *Prochlorococcus* genomes were also used as outgroup. Proteins were concatenated and subsequently aligned among the different genomes. A total of 122 conserved genes were found in all genomes (based on the TIGR database) and were used to create the reference phylogeny of *Synechococcus* (Supplementary Table S2). The alignment was performed using Kalign (Lassmann and Sonnhammer, 2005) and trimmed using trimal (Capella-Gutiérrez et al., 2009) using default parameters. A maximum-likelihood tree was constructed using FastTree2 (Price et al., 2010) using a JTT+CAT model, a gamma approximation and 100 bootstrap replicates.

### Single Gene Trees

16S rRNA *Synechococcus* sequences were aligned using MUSCLE (Edgar, 2004) and maximum likelihood 16S rRNA trees were constructed with MEGA7 using a gamma distribution and 100 bootstraps and considering a Tamura-Nei model (Tamura and Nei, 1993) with partial deletion, 95% of site coverage cut-off and Nearest-Neighbor-Interchange ML Heuristic Method (Kumar et al., 2016). RuBisCo small and large subunit trees were constructed using MEGA7 using a JTT model, a gamma approximation and 100 rapid bootstraps.

### Metagenomic Fragment Recruitment

Recruitments were performed against several publicly available freshwater and brackish metagenomes using BLASTN (Altschul et al., 1997). A hit was considered only when it was at least 50 nucleotides long, with >95% identity and an *e*-value of <= 1e^-5^. These hits were used to compute the RPKG (reads recruited per Kb of genome per Gb of metagenome) values that provide a normalized number comparable across metagenomes from different locations. All metagenomic data sets used in this work are publicly available: Tous reservoir (SRR4198666 and SRR4198832), Amadorio reservoir (Ghai et al., 2014), Lake Lanier (Oh et al., 2011) (SRR947737, SRR948334, SRR948334) and Dexter reservoir (SRR3184716). Two southwestern Mediterranean samples from TARA oceans consortium database (Sunagawa et al., 2015) were also included in the analysis (ERR315856 and ERR315857).

### Flow Cytometry Counts and Bacterioplankton Size Spectrum Determination

Water samples were fixed *in situ* with a paraformaldehyde:glutaraldehyde solution to a final concentration in the sample of 1%: 0.05% (w/v) (Marie et al., 1997). For flow cytometry identification and counting of the photosynthetic microbes and bacterioplankton, a Coulter Cytomics FC500 flow cytometer equipped with an argon laser (488 excitation), a red emitting diode (635 excitation), and five filters for fluorescent emission (FL1-FL5) was used. Cytometric parameter settings were as follows: FSC (550), SSC (390), FL1 (600), FL2 (670), FL3 (670), FL4 (620), and FL5 (700). Side scattering (SSC) was used for measuring size. Pcy enumeration was performed with discrimination by FL4, whereas for bacterioplankton identification, after 1h Sybr Green-I staining, discrimination was made by fluorescence in FL1 (Veldhuis and Kraay, 2000). Analyses were run for 120 s at the highest single flow rate of 128 μL min^-1^ of our machine. Abundance of each population was calculated according to the formula: *N* = (*n* × 1,000)/*q* × *t*, where *q* is the flow rate (microliter per minute), *t* is the length (minutes) of the data acquisition, *n* is the number of events counted by the flow cytometer, and *N* is the number of cells per milliliter. Flow rate was obtained gravimetrically considering the processed volume. Data were collected with the Beckman Coulter acquisition software for acquisition “CXP Version 2.2 Acquisition” and the analysis of the data was performed using Beckman Coulter analysis software for analysis “CXP Version 2.2 Analysis.”

### Microscopy and FISH Counts

For microscopic counts of picoplankton, water samples were fixed *in situ* with a paraformaldehyde:glutaraldehyde solution to a final concentration in the sample of 1%: 0.05% (w/v) (Marie et al., 1997). Once in the laboratory, subsamples of 5–10 mL were filtered through 0.2 μm pore size black filters (Nuclepore, Whatman). For quantification, a section of the filter was stained with 4′,6-diamidino-2-phenylindole (DAPI) (Porter and Feig, 1980) (SIGMA) and counted with an inverted Zeiss III RS epifluorescence microscope (1250×, resolution 0.02857 lm/pixel) using a G365 exciting filter, LP420 suppression filter for blue light and G546 exciting filter, LP590 suppression filter for green light (MacIsaac and Stockner, 1993). Autotrophic cells were identified by chlorophyll autofluorescence under green light.

For FISH detection of the specific *Synechococcus* sp. group isolated from Tous reservoir, water samples were fixed *in situ* with paraformaldehyde to 2% final concentration, then filtered within the next 2 h onto white polycarbonate filters (0.2 μm pore size, Whatman). Filter sections were stained with the different oligonucleotide probes and with DAPI, and then mounted for microscopic evaluation. We primarily used a previously described general probe “Syn405” ^1^ (West et al., 2001; Arnosti et al., 2012) (5′-AGAGGCCTTCATCCCTCA-3′) for total *Synechococcus* counts. For specific *Synechococcus* sp. identification we designed a specific probe using the PRIMER3 tool (Rozen and Skaletsky, 1999). This new probe (“SynTo,” ‘5′-TGGCCCAGCAGAGCGCTTTC-3′’) was double labeled with the indocarbocyanine dye Cy3 and Cy5 (Thermo Scientific, Waltham, MA, United States). These probes were checked for specificity using the Probe Match RDP (Cole et al., 2009) RDP Release 11, Update 5: September 30, 2016 (3,356,809 16S rRNA database^2^). No specific experimental test with *Synechococcus* pure cultures were performed with the probe “SynTo” but RPD probe match show extremely high specificity for genus GPIIa (family II) of cyanobacteria and specifically hit to *Synechococcus* RCC307 (CT978603). The FISH protocol was performed as previously described (Behnam et al., 2012). Hybridization conditions for the probe were adjusted by formamide (VWR BDH Prolabo) 20-40 % series applied to different subsamples. Each filter was cut in sections to be used with different probes. For cell-wall permeabilization, each filter section was incubated in a lysozyme solution for 30 min at 37°C. The sections were washed with MQ water and 80% ethanol for 3 min. Subsequently, the filter sections were placed on glass slides and covered with 20 ml of hybridization solution, which contains 5 M NaCl, 1M Tris-HCl, 10% sodium dodecyl sulfate, and 35% formamide; next 2 μl of “SynTo” probe were added and the filter section was incubated at 46°C for 90 min in an equilibrated chamber (Eppendorf thermomixer for slides). After hybridization, the sample was transferred into a washing solution containing 1 M Tris-HCl, 0,5 M EDTA, 10% sodium dodecyl sulfate, 5 M NaCl and 10 mg/ml DAPI, then incubated twice at 46°C for 15 min. After incubation, the sections were rinsed in 80% ethanol for a few seconds and placed in MQ water for 5 min. After complete drying each section was mounted in oil for microscopic determinations. Absolute densities of hybridized *Synechococcus* were calculated as the product of their relative abundances on filter sections (percentage of DAPI-stained objects) and the DAPI-stained direct cell counts. A minimum of 500 DAPI and probe-stained cells were measured per sample. Images from FISH determinations were analyzed using the NIH IMAGEJ software to determine cell dimensions^3^.

## Results and Discussion

### Environmental Parameters of Tous Reservoir

At the time of sampling (February 20th, 2015) the water column of the freshwater (conductivity <400 μS⋅cm^-1^) Tous reservoir was mixed and consequently both samples, from 12 and 25 m depth, had mostly similar physicochemical features (Supplementary Figure S1). Water had a relatively high transparency (Secchi Disk depth of nearly 8 m) as corresponds to an oligotrophic system, with maximum chlorophyll-a concentration lower than 2 μg⋅L^-1^ found at medium depths (10–15 m). Phosphorus appeared as the limiting nutrient, since inorganic nitrogen, mainly nitrate, was far more abundant (Supplementary Figure S1). The determinations of abundances by flow cytometry showed that *Synechococcus* cells were distributed throughout the water column at moderate abundances of around 2 × 10^4^ cells⋅mL^-1^, whereas heterotrophic bacteria reached around 6 × 10^5^ cells⋅mL^-1^ at all tested depths.

### Community Structure of the Tous Metagenome Based on 16S rRNA Reads

The taxonomic composition based on 16S rRNA coding raw read fragments found in the Tous reservoir shows that taxa are present in similar proportions to the ones found in Amadorio (10 m, 0.22 μm collected sample), the closest freshwater water body to Tous (Ghai et al., 2014). In comparison to Lake Lanier, a similar temperate reservoir (Georgia, United States) (Supplementary Figure S2), some differences could be detected such as the higher numbers of Actinobacteria (nearly 40% in Tous 12 m in contrast to the 20% found in Lanier S2 sample (taken in July at 5 m depth), Bacteroidetes (around 10–15% in Tous samples compared to <5% in Lake Lanier) and Alphaproteobacteria (20% in Tous samples vs. <10% in Lake Lanier). On the other hand, Verrucomicrobia comprise 14% of the total 16S rRNA reads in Lake Lanier samples, compared to the 7–8% in Tous and Amadorio. Gammaproteobacteria were also present at higher proportions in Lake Lanier. Cyanobacteria comprise significant relative numbers according to the 16S rRNA classification in freshwater lakes like Albufera, Lake Lanier, Mendota (summer), Trout, Vattern, Amadorio (Ghai et al., 2014) and Tous 12 and 25 m samples, ranging between 4 and 10% in these freshwater bodies. Also, there was an important increase in Lanier 16S rRNA cyanobacterial reads in the Lake Lanier S2 sample reaching 14% of the reads compared to the other sites.

### Genome Reconstruction and Phylogenomics of *Synechococcus* sp. Assembled Genomes

By a metagenomic approach we were able to reconstruct genomes of novel freshwater *Synechococcus.* Genomes derived from metagenomes are always composites of several clones that coexist in aquatic environments (Rodriguez-Valera et al., 2009; Kashtan et al., 2014). However, the quality of the assembly (long contigs) and relatively easy binning, points to all the clones belonging to a single species.

A total of 1.798 Mb of assembled composite genome were retrieved for this new freshwater *Synechococcus* sp. (hereafter referred to as *Synechococcus* sp. Tous). A genome completeness of 97.14% and genome size estimation of 1.85 Mb were obtained utilizing a set of 35 essential genes (Raes et al., 2007), whereas 74.77% and 2.4 Mb were estimated using a set of 112 essential genes (Albertsen et al., 2013), respectively. A total of 1959 proteins were predicted and annotated in this genome.

Lake Lanier, a freshwater reservoir on Chattahoochee River in Georgia (United States) extends over a surface of 150 km^2^. It is larger than the Tous reservoir, with a maximum depth of 78 m and a maximum volume of around 1294 Hm^3^, but presents a much smaller catchment area than Tous (2700 km^2^). Given the high recruitment (at >95% sequence identity in nucleotide comparisons) of the *Synechococcus* sp. Tous genome in the Lake Lanier metagenomes we attempted to assemble the relative present there as well. *Synechococcus* sp. Lanier genome reconstruction provided 1.475 Mb in 79 contigs. A total of 1619 proteins were predicted and annotated for *Synechococcus* sp. Lanier. The completeness of 40 and 50.45% according to Raes et al. (2007) and Albertsen et al. (2013) was much lower, likely due to the lack of the large contigs provided by fosmid libraries that were not constructed for Tous. For example, the Lanier genome is missing 30 genes from one of the ribosomal proteins cluster and the rRNA operon.

**FIGURE 1:**
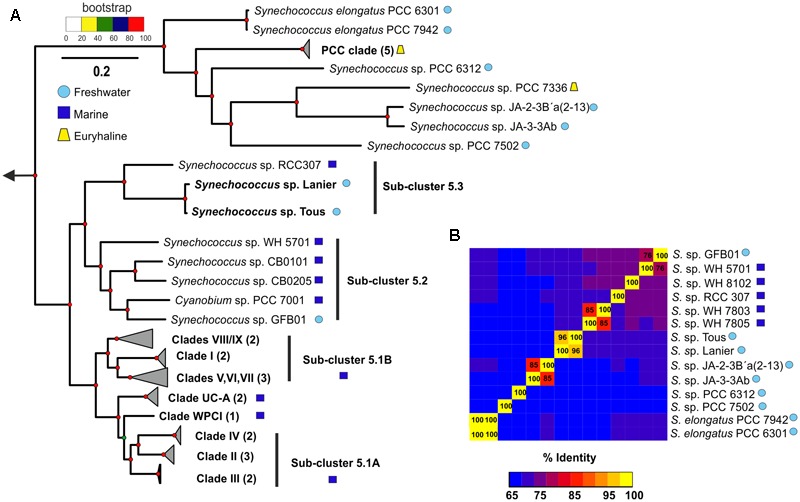
**(A)** Phylogenomics of the genus *Synechococcus* sp. One hundred twenty-two conserved genes were used to generate a maximum-likelihood phylogenetic tree with marine, freshwater and the novel *Synechococcus* sp. representatives. Three *Prochlorococcus* genomes were used as an outgroup. **(B)** ANI (average nucleotide identity) between the closest marine and freshwater representatives to the novel *Synechococcus* sp. Tous/Lanier.

To infer the phylogenetic relationships of the new Tous and Lanier *Synechococcus* sp. genomes, a phylogenomic tree consisting of a concatenation of 122 common genes (Supplementary Table S1) of marine and freshwater *Synechococcus* sp. was constructed, using *Prochlorococcus* as an outgroup (**Figure 1A**; for a detailed tree see Supplementary Figure S3). The resulting tree recapitulates the phylogeny of the entire *Synechococcus* genus with marine and freshwater representatives. A 16S rRNA tree is also provided in Supplementary Figure S4. The phylogenetically closest species to these new freshwater genomes, as expected from the similarities detected in the metagenomic contigs (see above), was the marine *Synechococcus* sp. RCC307, a representative of the marine sub-cluster 5.3 isolated from a 15 m depth sample from off-shore Western Mediterranean waters. Classically, the sub-cluster 5.1 has been considered exclusively marine (Six et al., 2007; Dufresne et al., 2008; Scanlan et al., 2009), while sub-cluster 5.2 contains phycocyanin-containing euryhaline strains widely distributed in coastal areas and estuaries and sub-cluster 5.3 contains marine (*Synechococcus* sp. RCC307) and other less studied phycoerythrin-containing strains (Scanlan et al., 2009). Our two freshwater genomes clearly associate with sub-cluster 5.3 while the freshwater *Synechococcus* sp. GFB01 (Guimarães et al., 2015) associates with sub-cluster 5.2, which indicates that these two sub-clusters contain strains of both marine and non-marine origin, including freshwater. Actually, a 16S rRNA study of halotolerant strains showed that they also belonged to the 5.3 sub-cluster (Callieri et al., 2013).

**FIGURE 2:**
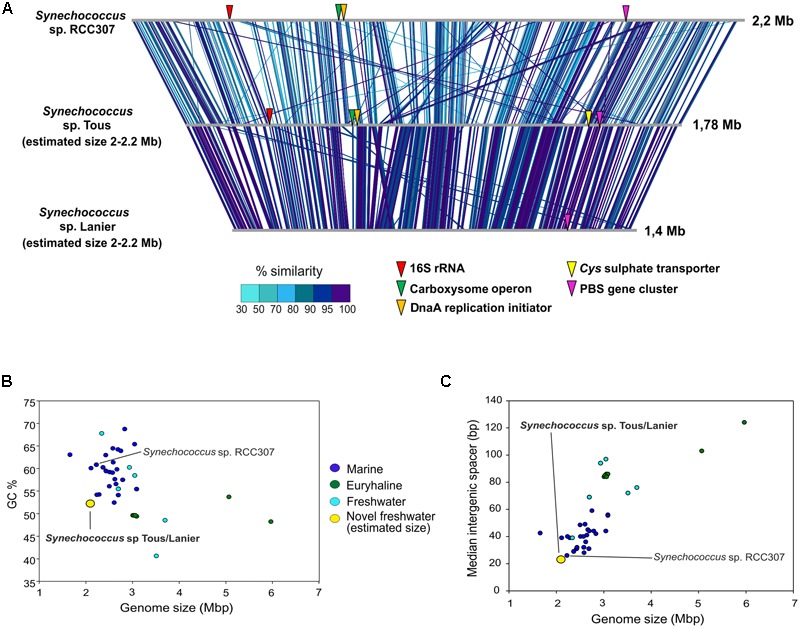
**(A)** Genomic comparison between marine *Synechococcus* sp. RCC307 and the novel freshwater assembled genomes from Tous and Lanier. Comparison made with BLASTN with 50 bp alignment length and >70% similarity. 16S rRNA, DnaA replication initiator, carboxysome operon, Cys sulfate transporter and PBS genes are indicated in the genomes. **(B)** Genome size (Mbp) vs. GC content of all marine, freshwater and euryhaline *Synechococcus* sp. sequenced genomes. **(C)** Genome size (Mbp) vs. median intergenic spacer (bp) of all *Synechococcus* sp. genomes. Estimated genome size was used for the novel freshwater *Synechococcus* sp.

The contigs obtained from the metagenomic reconstruction showed remarkable synteny with the *Synechococcus* sp. RCC307 genome. Therefore, we used this genome as a reference for the virtual reconstruction of the novel genomes (**Figure 2A**). ANI between *Synechococcus* sp. from Tous and Lanier was 96%, which indicates that the two assembled genomes are the same species within a novel *Synechococcus* sp. (**Figure 1B**). Despite the proximity and closeness of these two genomes to *Synechococcus* sp. RCC307 in the phylogenomic tree (**Figure 1A**) and their localized (at least) synteny (>70% similarity and 50 bp alignment length) (**Figure 2A**), ANI was only ca. 68% among both genomes and the marine isolate (**Figure 1B**). ANI was also performed against other marine and freshwater representatives, finding in all cases less than 70% among these novel species and the rest of the known *Synechococcus.* However, 16S rRNA showed high values over 97% similarities with several *Synechococcus* species confirming the lack of discriminatory power of this marker in Pcy in general. Taken together, both these assembled genomes (even considering their incompleteness) fall at the lower size range of all known *Synechococcus* genomes that are extremely variable in size, ranging from ca. 2 to 6 Mb) (**Figures 2B,C**). They have the smallest median intergenic spacer lengths of all known *Synechococcus* genomes so far (**Figure 2C**), suggesting a streamlined genome.

### Abundance of the Novel *Synechococcus* Genomes

**FIGURE 3:**
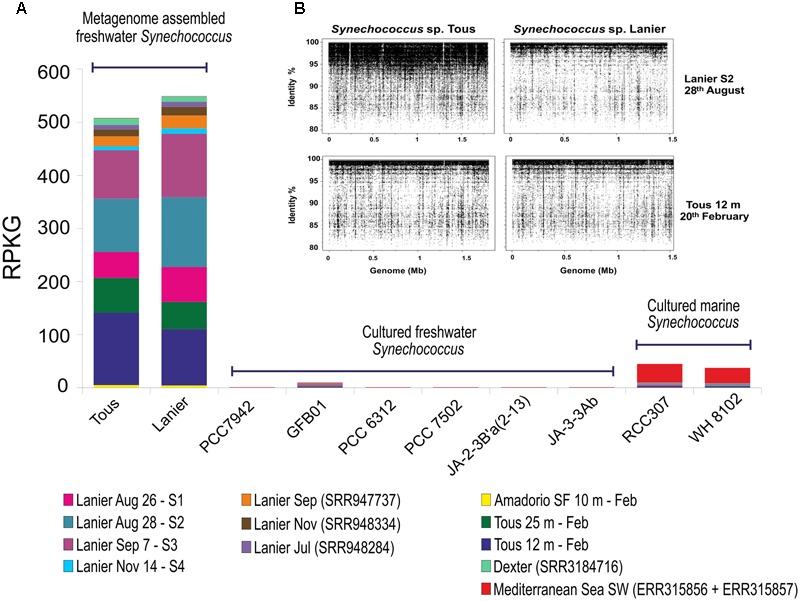
**(A)** Fragment recruitment of novel and reference *Synechococcus* sp. Genome abundance (expressed as RPKG, reads per Kb of genome per Gb of metagenome) along different freshwater metagenomes and two marine metagenomes. Only datasets with >5 RPKG values for the novel freshwater *Synechococcus* sp. were included in this analysis. Only hits with ≥95% identity, ≥50 bp alignment length were considered. Sep, September; Nov, November; Feb, February; Jul, July; Aug, August; SF, small fraction. **(B)** Metagenomic recruitment of *Synechococcus* sp. Tous and Lanier assembled genomes against Lanier S2 sample (5 m August 28th, 2011) (I) and Tous 12 m February 2014 (II) metagenomes. A 95% identity cut-off and 50 bp read length on each metagenome were used as restrictive parameters.

To assess the distribution of the novel assembled *Synechococcus* sp. in other freshwater and brackish habitats, fragment recruitment was carried out against different metagenomes available from water bodies of different salinities (see Materials and Methods). The only available reference freshwater *Synechococcus* genomes, *Synechococcus* sp. GFB01 (Guimarães et al., 2015), *S. elongatus* PCC7942 (Holtman et al., 2005), Yellowstone representatives *Synechococcus* JA-3-3Ab and JA-2-3B’a(2-13) (Bhaya et al., 2007) and *Synechococcus* sp. PCC7502 and PCC6312 (Shih et al., 2013) were used for recruitment together with the phylogenetically closest marine representative *Synechococcus* sp. RCC307 and another marine *Synechococcus* sp. WH8102 (Scanlan et al., 2009). The freshwater metagenomes which provided significant recruitment values higher than 5 RPKGs for the *Synechococcus* sp. Tous and Lanier genomes are shown in **Figure 3A**. Tous and Lanier reservoirs from which the genomes were assembled showed, as might be expected, the highest number of RPKG (reads per Kb of genome per Gb of metagenome at >95% identity) (**Figure 3**). Both reconstructed *Synechococcus* spp. genomes clearly dominate Lanier and Tous metagenomes, which is reflected by the fragment recruitment identity values between 95 and 100% (**Figure 3B**). Both temperate reservoirs are located in very similar latitudes and altitudes (39°N and 64 m above average sea level for Tous reservoir and 34°N and 326 m for Lake Lanier) but are very distant geographically and located on different continents. Highest abundance values for both assembled genomes were found in Tous 12 m and Tous 25 m samples. In the Amadorio reservoir, that is only 100 km from Tous, 5–10 times lower values were found. Abundance in Lake Lanier samples used for assembly was comparable to that in Tous (**Figure 3A**). The novel genomes were also found in one metagenomic sample taken during a cyanobacterial bloom in Dexter reservoir (Oregón, United States). Only very low levels were found in Sparkling lake (Martinez-Garcia et al., 2012) and Albufera lagoon (Ghai et al., 2012). This suggests that these novel *Synechococcus* sp. could be widespread in many other temperate freshwater reservoirs with similar environmental parameters, being globally distributed at similar latitudes as has been previously described for marine *Prochlorococcus* and *Synechococcus* (Flombaum et al., 2013). The absence of the novel *Synechococcus* spp. in cold lakes with freezing cycles during the winter season, like Yellowstone, Lake Mendota, Swedish lakes (Erken, Ekoln and Vattern) or the Laurentian Great Lakes (Michigan and Ontario) also supports its temperate specialization. Low recruitment values were also found in Lake Gatun, Trout, Damariscotta, Lake Houston and the Amazon River. Interestingly, no other freshwater *Synechococcus* genomes recruited significant amounts from any of these metagenomes. Neither marine *Synechococcus* sp. RCC307 nor WH8102 were significantly detected (less than 2 RPKG) in the freshwater or brackish datasets tested. In the two marine metagenomes from the TARA oceans database, one from a 4 m sample in the southwest Mediterranean Sea (ERR315856) and another from 40 m depth from the same location (ERR315857), marine *Synechococcus* sp. RCC307 and WH8102 recruited significantly (20–25 RPKG) supporting their broad distribution in marine habitats, contrastingly to their absence in freshwater. The assemblies from Tous and Lanier did not recruit at all from the marine samples. These data suggest that the two assembled genomes represent a species of *Synechococcus* that is widely distributed in fresh water bodies at temperate latitudes and are among the abundant photosynthetic bacteria in these habitats.

**FIGURE 4:**
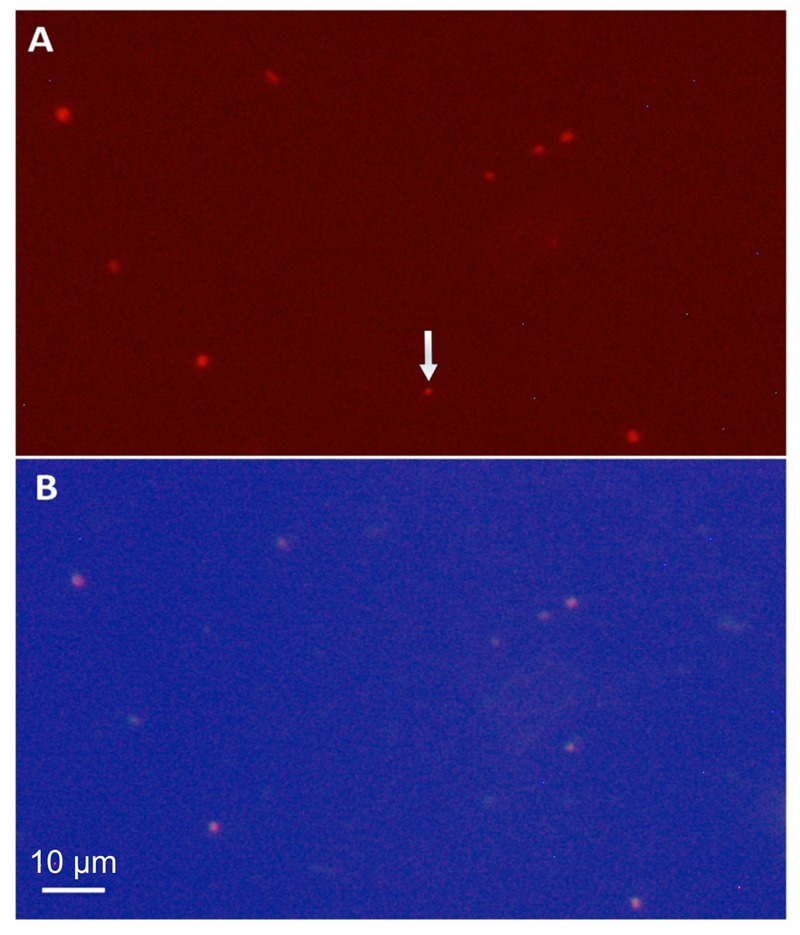
Photomicrographs (1250×) of a 12 m depth sample from Tous reservoir. **(A)** Phycoerythrin fluorescence under a green filter showing all picocyanobacteria in the sample. **(B)** FISH microphotograph of the same microscopic field stained with the Cy3, Cy5 fluorescence labeled rRNA-targeted probe specific for *Synechococcus* sp. Tous (“SynTo”). Note that all picocyanobacteria except that marked by the arrow are targeted by the specific “SynTous” probe. Scale bars represent 10 μm.

The abundance of members of the species represented by the assembled genomes in the Tous reservoir was confirmed by FISH (**Figure 4**). A total of 87.8% of the cells that hybridized to the general *Synechococcus* probe Syn405 also did with the specific probe for *Synechococcus* sp. Tous “SynTo.” Cells had rather small sizes (0.987 ± 0.139 μm lengths and 0.723 ± 0.119 μm width). From the general description of the genome provided below and the microscopic image we propose the name *Ca. Synechococcus lacustris* for the microbes identified by these two genomes and microscopically detected by FISH.

### PBS Gene Cluster of the Novel Freshwater *Synechococcus* spp.

**FIGURE 5:**
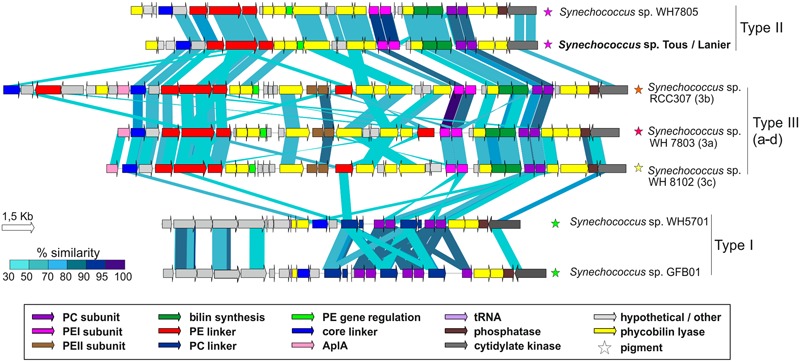
Structure and similarity of the PBS gene cluster among marine and Tous/Lanier freshwater *Synechococcus* sp. Comparison made with TBLASTX with >30% similarity hits and 150 bp alignment lengths. PC, Phycocyanin; PE, Phycoerythrin; AplA, Allophycoyanin-like protein; Phycobilin lyases, CpeY, CpeZ, CpeF, CpeS, CpeT, CpeU, RpcG, RpcE, RpcE, RpcF, RpcT; Bilin synthesis, pebA, pebB.

As seen in **Figure 5**, the novel *Synechococcus* sp. Tous/Lanier genome presents a highly similar PBS gene cluster structure to *Synechococcus* sp. WH7805, isolated in the Sargasso Sea (Six et al., 2007). The lack of PEII subunit in WH7805 and the new genomes points toward the novel *Synechococcus* spp. having type II pink pigmentation, being ecotypes adapted to low light ranges, typically found, for example, in deep marine oligotrophic waters (Scanlan et al., 2009; Callieri et al., 2012). Freshwater *Synechococcus* sp. GFB01 presents five PC subunits, one more as compared to the type I marine homolog WH5701. The only differences observable in the comparison of type II pigment containing strains WH7805 and the novel freshwater species is the lack of the low molecular weight tyrosine-phosphatase and two hypothetical proteins, which could be unique characteristics in the freshwater type II pigmentation strains. In any case, for each pigment type the structure of the PBS gene cluster is maintained in both freshwater and marine genomes.

### Features of the *Synechococcus* sp. Assembled Genomes That Support Their Freshwater Specialization

RuBisCo large and small subunits found in the *Synechococcus* sp. Tous assembled genome were phylogenetically close to those found in *Paulinella chromatophora* (Supplementary Figures S5, S6), a freshwater photosynthetic amoeba with chloroplasts originating from a photosynthetic symbiont related to *Synechococcus* (Marin et al., 2007). In addition, 30S ribosomal proteins and some photosynthetic proteins like photosystem I subunit VII and photosystem II D2 protein gave very high similarities (over 98%) to their homologs in the amoeba chloroplasts. That a freshwater amoeba receives an endosymbiont from a freshwater *Synechococcus* makes eco-evolutionary sense (Blank and Sanchez-Baracaldo, 2010).

As expected, the *ntc*A transcriptional regulator and nitrogen regulatory p-II protein common cyanobacterial genes were found in both Lanier and Tous genomes. Both *Synechococcus* sp. are able to obtain urea from the environment since the *urt*ABCDE transport system and urease cluster *ure*ABCDEFGH were detected in both assembled genomes. Glutamine synthetases (GlnA and GlnIII), which are missing in *Synechococcus* sp. RCC307 (but are present in other marine strains), were also found in both freshwater *Synechococcus* spp. Ammonia is the preferred N source in Pcy, as it can be incorporated easily to the amino acid glutamine and later on glutamic acid (Scanlan et al., 2009). Two *amt*-family ammonia channel proteins with small similarities (<60%) to other marine *Synechococcus*, but closer to the freshwater cyanobacteria *Cyanobium* sp. CACIAM14 (at 89% similarity), *Cyanobium gracile* PCC6307 or *Synechococcus* sp. GFB01, were detected in both genomes.

A common freshwater transporter Cys (specific for sulfate transport) which is absent in marine but present in freshwater cyanobacteria (see below) was found in the genomes. *Synechococcus* contain two sulfate permeases known as *sul*P (Price et al., 2004) in their genomes to acquire sulfur. A different sulfate ABC transport system has been described for some freshwater cyanobacteria like *S. elongatus* PCC7942. It consists of three *Cys* genes (*Cys*W, *Cys*T, and *Cys*A) which form the membrane components and the *sbp*A gene, that codes for a periplasmic binding protein (Laudenbach and Grossman, 1991). *S. elongatus* PCC7942 contains a gene cluster with two subunits of each of these four genes; mutation of *Cys*T, *Cys*W or *Cys*A genes in this strain led to no sulfate uptake and no growth when the latter was used as the sole sulfur source (Laudenbach and Grossman, 1991). Supplementary Figure S7 shows the comparisons made between *Synechococcus* sp. from Tous and the freshwater *Cyanobium* sp. CACIAM 14, *Cyanobium gracile* PCC6307, *Synechococcus* sp. GFB01 and the marine representative RCC307 showing a marked synteny among the freshwater ones and absence in the marine representative. This sulfate transport system is also preserved among many other freshwater Pcy, even filamentous genera like *Tolypothrix, Fischerella*, or *Scytonema.* The absence of this transporter in marine *Synechococcus*, could be explained by the high sulfate concentrations in the ocean which do not require the presence of these high affinity ABC sulfate transporters.

Freshwater cyanobacteria generally appear to have a requirement for zinc detoxification while some marine *Synechococcus* and *Prochlorococcus* do not seem to have it (Blindauer, 2008). Among the different transport systems detected in *Synechococcus* sp. Tous genome, we found ZnuC, ZnuB zinc/manganese and ZupT (IPR023498) zinc transporters. Surprisingly, the latter ZupT transporter was not found in marine or other freshwater cyanobacteria. Furthermore, this gene had highest similarities (<65 of relative abundance) to some found in green-sulfur photosynthetic or sulfur-reducing bacteria like *Chlorobium, Desulfobacter*, or *Desulfuromonas*.

Previous studies comparing the isoelectric point proteome of halophilic, marine and freshwater bacteria have shown that salinity adaptation positively correlates with a protein shift toward acidity in halophilic bacteria (Bardavid and Oren, 2012). Instead, a typical bimodal pattern is observed in marine and more pronouncedly in freshwater microbes, the latter presenting a lower peak in acidic proteins and a higher peak in basic proteins compared to marine representatives. We performed a whole proteome isoelectric point profile comparison between marine and freshwater *Synechococcus* (Supplementary Figure S8A). As expected, while marine *Synechococcus* showed a high percentage of acidic proteins, which presumably helps them adapt to their saline environment, improving the hydration sphere of the proteins, they also displayed a very low fraction of basic proteins. In contrast, freshwater *Synechococcus* sp. Tous, Lanier and GFB01 exhibited a lower peak of acidic proteins and a higher peak of basic amino acids. Freshwater *S. elongatus* PCC7942 showed both peaks of acid and basic proteins, consistent with its ability to tolerate different pH and salinity ranges (Billini et al., 2008).

We also compared the isoelectric points (pI) for all type of annotated transporters with transmembrane domains like ABC-type, permeases, efflux systems and also membrane proteins expecting that these exposed proteins would have the effect of salt amplified. Indeed, (Supplementary Figure S8B), marine *Synechococcus* showed a higher percentage of acidic transporters and membrane proteins and less basic transporters compared to freshwater genomes. Differences were even sharper when we considered exclusively those transporters and membrane proteins with pI ranging from 3.5 to 6.5 and from 8.5 to 12.5 (Supplementary Figure S8C). These pI plots further support that the novel *Synechococcus* sp. assembled genomes are specialized to live in freshwater.

Other mechanisms marine microbes use to deal with salinity adaptation rely on the active transport of nutrients in exchange of sodium, via co-transport symporters and translocation systems (Ventosa et al., 1998; López-Pérez et al., 2013). As expected, *S. elongatus* PCC7942, *Synechococcus* sp. GFB01 and the novel freshwater genomes did not contain any of these transporters. Sodium/proton antiporters were detected in both marine and freshwater strains, but especially in freshwater *S. elongatus* PCC7942 there are several copies of these transporters, as previously described (Billini et al., 2008). The accumulation of potassium as pH regulator through KefB and Trk transporters and the presence of sodium/proton antiporters, which couple with the maintenance of pH homeostasis in cells, have been described previously as strategies of salt adaptation (Mongodin et al., 2005). Either one or both potassium transporters were detected in both marine and freshwater *Synechococcus*. On the other hand, 11, 9, and 16 sodium dependent transporters of C-4 dicarboxylates osmoprotectants like glycine betaine and the amino acids proline or alanine were found in the marine *Synechococcus* sp. RCC307, WH8102 and WH7805, respectively (Scanlan et al., 2009), but none in the freshwater genomes. Sodium dependent transporters for sulfate, calcium, bicarbonate or bile acids were also found in marine genomes but not in the freshwater ones.

## Conclusion

The scarcity of sequenced freshwater *Synechococcus* sp. genomes complicates an update of the current classification of the genus, particularly sub-clusters 5.2 and the less studied 5.3, both containing marine, euryhaline and freshwater species. In this manuscript we describe two freshwater genomes that belong to sub-cluster 5.3. ANI between the novel freshwater species and the rest of the marine or freshwater genomes never reached 70%, which confirms the novelty of the new freshwater genomes. Their bona fide freshwater adaptation is clear from several lines of evidence, from metagenomic recruitment to the pI of the proteome or the presence/absence of specific transporters.

The massive predominance of this single species in these two lakes located in two different continents and more than 7000 km apart indicates a potentially widespread distribution in temperate latitudes. However, more metagenomics latitudinal gradients in distant lakes are required to establish their global distribution and habitat range. Interestingly, both freshwater *Synechococcus* spp. described here were not found in cold lakes with annual ice-in and ice-out freezing cycles regardless of their habitat type. Neither eutrophic reservoirs or oligo-mesotrophic Laurentian Great Lakes nor Swedish Lakes (Vattern and Erken) seem to be appropriate environments for the novel *Synechococcus* sp. This distribution supports their specialization in temperate oligo-mesotrophic environments, which is the case for both Tous reservoir and Lake Lanier. It is noteworthy that the genomes described here are the smallest freshwater *Synechococcus* discovered thus far (2.2 Mb of estimated genome size), which display a certain degree of streamlining as shown by their short mean intergenic spacer size (20 bp). These reduced and compact genomes would be an advantage in oligotrophic waters such as those in the seasonally stratified temperate lakes, decreasing cell size and increasing volume to surface ratio. Attempts to isolate microbes belonging to *Ca*. *S. lacustris* are underway.

## Data Accessibility

Tous reservoir metagenomic datasets have been deposited in the NCBI SRA database with BioProject number PRJNA342151 (SRR4198666 and SRR4198832). The assembled genomes *Synechococcus* sp. Lanier and *Synechococcus* sp. Tous have been deposited in the NCBI under Biosample identifiers SAMN05915837 and SAMN05915836, respectively. The cyanobacterial fosmids from Amadorio reservoir have been deposited to NCBI to the bioproject number PRJNA238866.

## Author Contributions

FR-V, AC, and PC-Y conceived this work. RG, AP and AC performed the sample collection and filtration. JH-M performed the DNA extraction. Analysis was carried out by PC-Y, A-BM-C, AP, and JH-M. AP and AC analyzed the sample properties. Manuscript was written by PC-Y, AC, RG, and FR-V. All authors read and approved the final manuscript.

## Conflict of Interest Statement

The authors declare that the research was conducted in the absence of any commercial or financial relationships that could be construed as a potential conflict of interest.

## Footnotes

1 http://probebase.csb.univie.ac.at/pb_report/probe/766

2 https://rdp.cme.msu.edu/index.jsp

3 http://rsb.info.nih.gov/ij/index.html
