# Supplementary material for: Novel Synechococcus Genomes Reconstructed from Freshwater Reservoirs
Source: Front Microbiol. 2017 Jun 21;8:1151. doi: 10.3389/fmicb.2017.01151 (PMC5478717; doi:10.3389/fmicb.2017.01151)
Supplement: Supplementary file 3 [file Presentation_1.PDF]

Supplementary Material

Novel and abundant *Synechococcus* sp. genomes assembled from freshwater reservoirs

Pedro J. Cabello-Yeves[1], Jose M. Haro-Moreno[1], Ana - Belen Martin-Cuadrado[1], Rohit Ghai [3], Antonio Picazo[2], Antonio Camacho[2] and Francisco Rodriguez-Valera[1*]

[1]Evolutionary Genomics Group, Departamento de Producción Vegetal y Microbiología, Universidad Miguel Hernández, San Juan de Alicante, 03550 Alicante, Spain, [2]Cavanilles Institute of Biodiversity and Evolutionary Biology, University of Valencia, Burjassot, E-46100 Valencia, Spain

[3]Institute of Hydrobiology, Department of Aquatic Microbial Ecology, Biology Center of the Academy of Sciences of the Czech Republic, České Budějovice, Czech Republic

*Correspondence: Francisco Rodríguez-Valera, E-mail: frvalera@umh.es

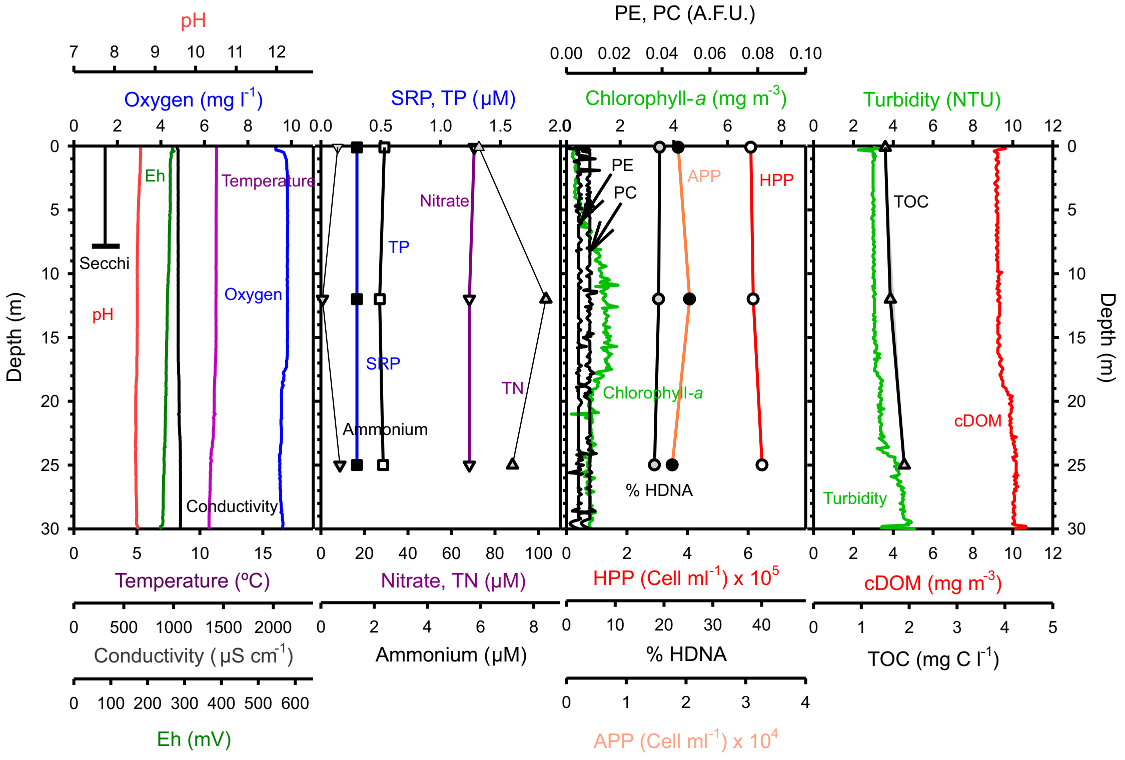

Supplementary Figure S1. Vertical profiles of the main environmental variables in Tous reservoir at the date of sampling (February 20th, 2015).

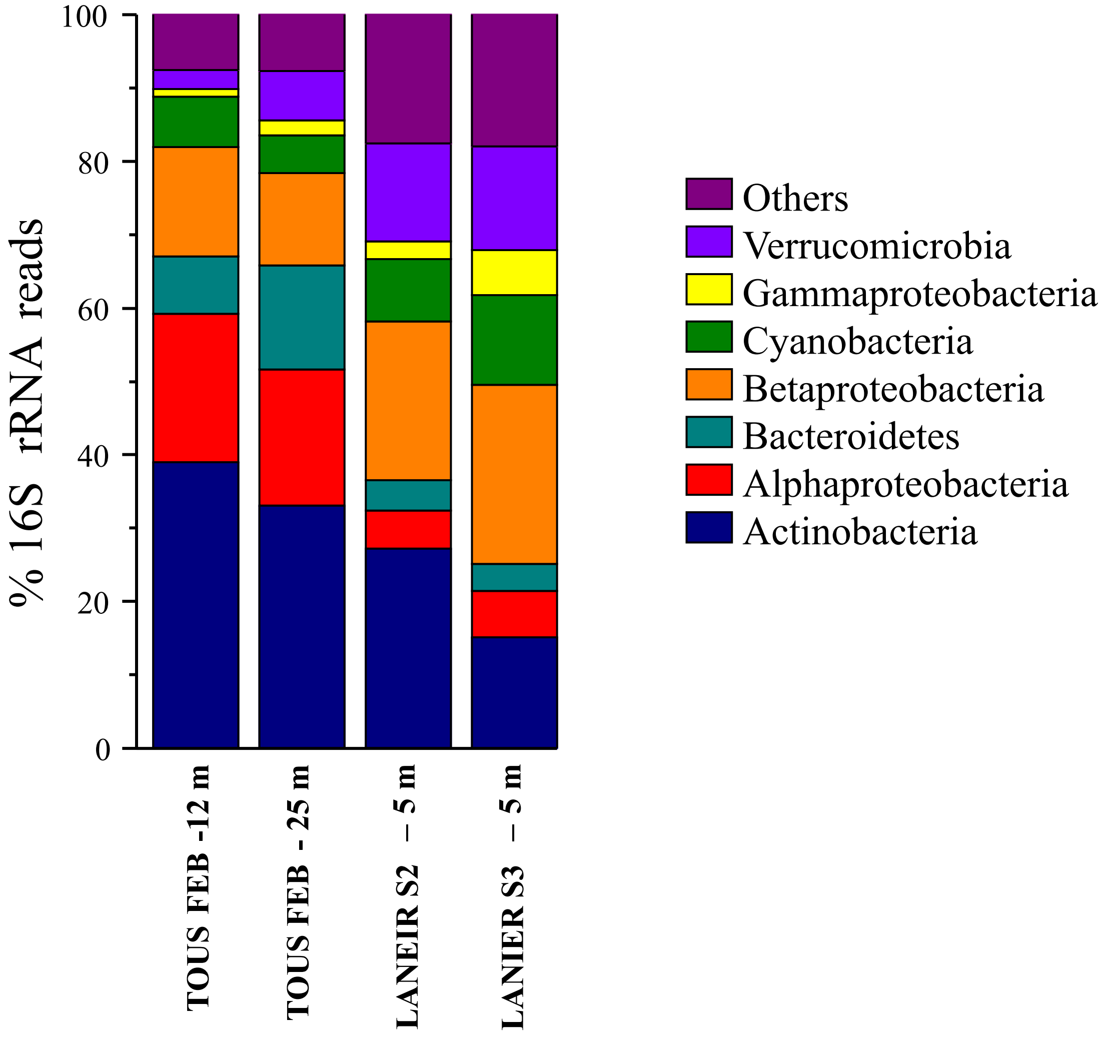

Supplementary Figure S2. 16S rRNA classification for Tous reservoir (12 and 25 m, February 20th, 2015) and Lake Lanier (S2: August 28th and S3: September 7th, 2009).

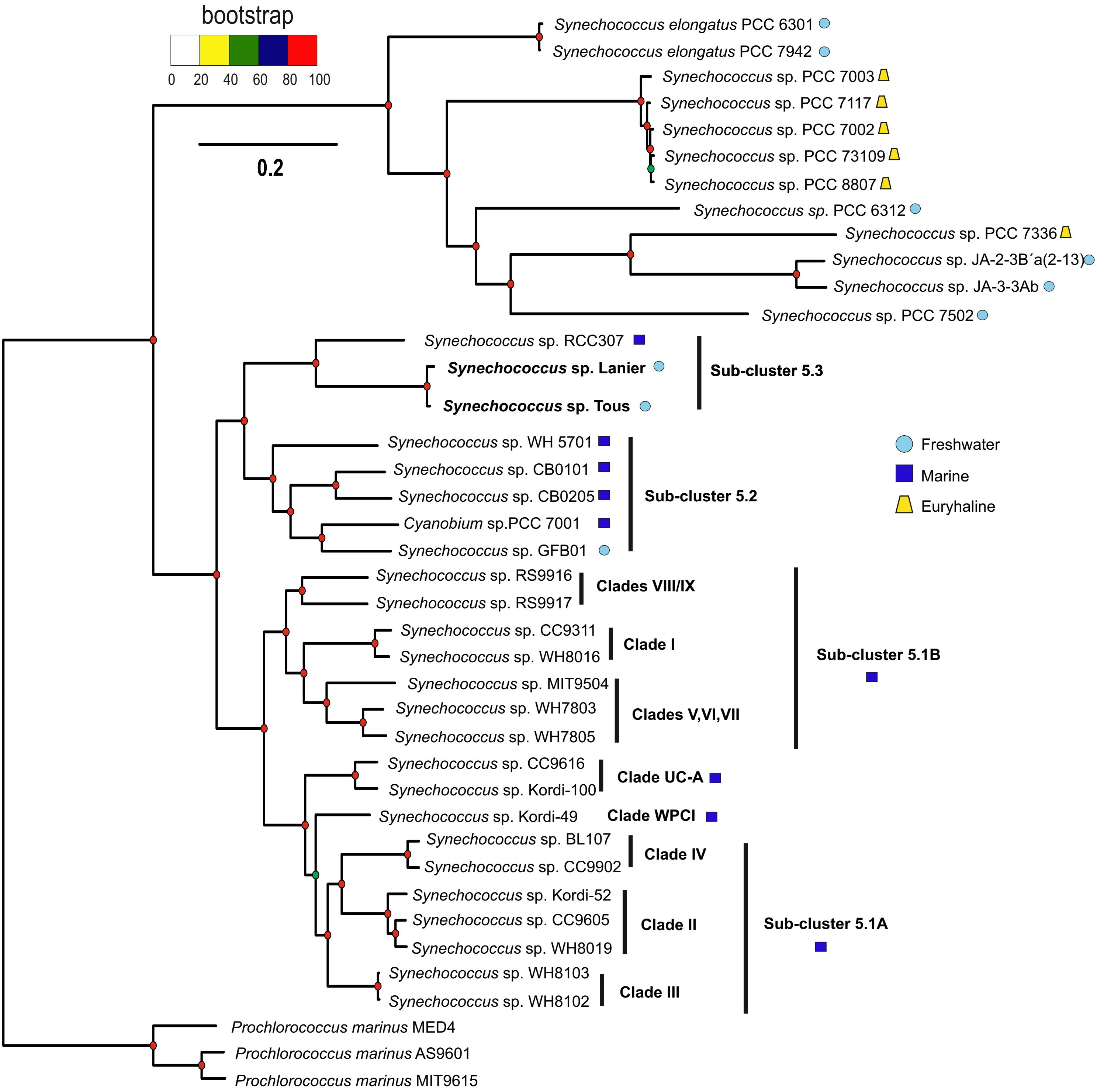

Supplementary Figure S3. Detailled phylogenomics of the genus *Synechococcus* sp. A total of 122 conserved genes were used to generate a maximum-likelihood phylogenomic tree with marine, freshwater and the novel *Synechococcus* sp. representatives. Three *Prochlorococcus* genomes were used as an outgroup.

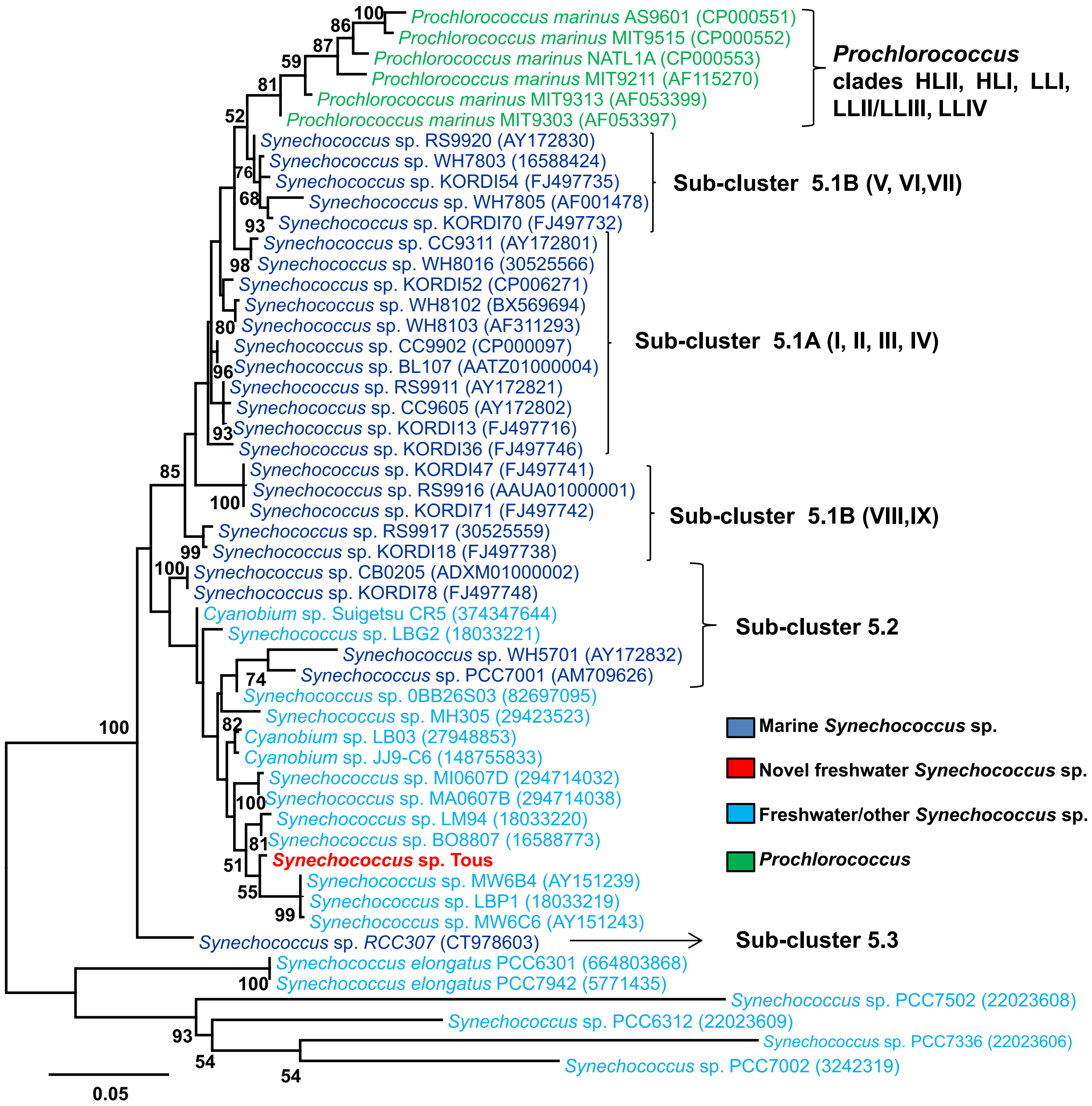

Supplementary Figure S4. Maximum likelihood 16S rRNA gene tree including marine, freshwater and the novel *Synechococcus* sp. *Cyanobium* and *Prochlorococcus* representatives are also included. Bootstrap values of > 50 % are shown.

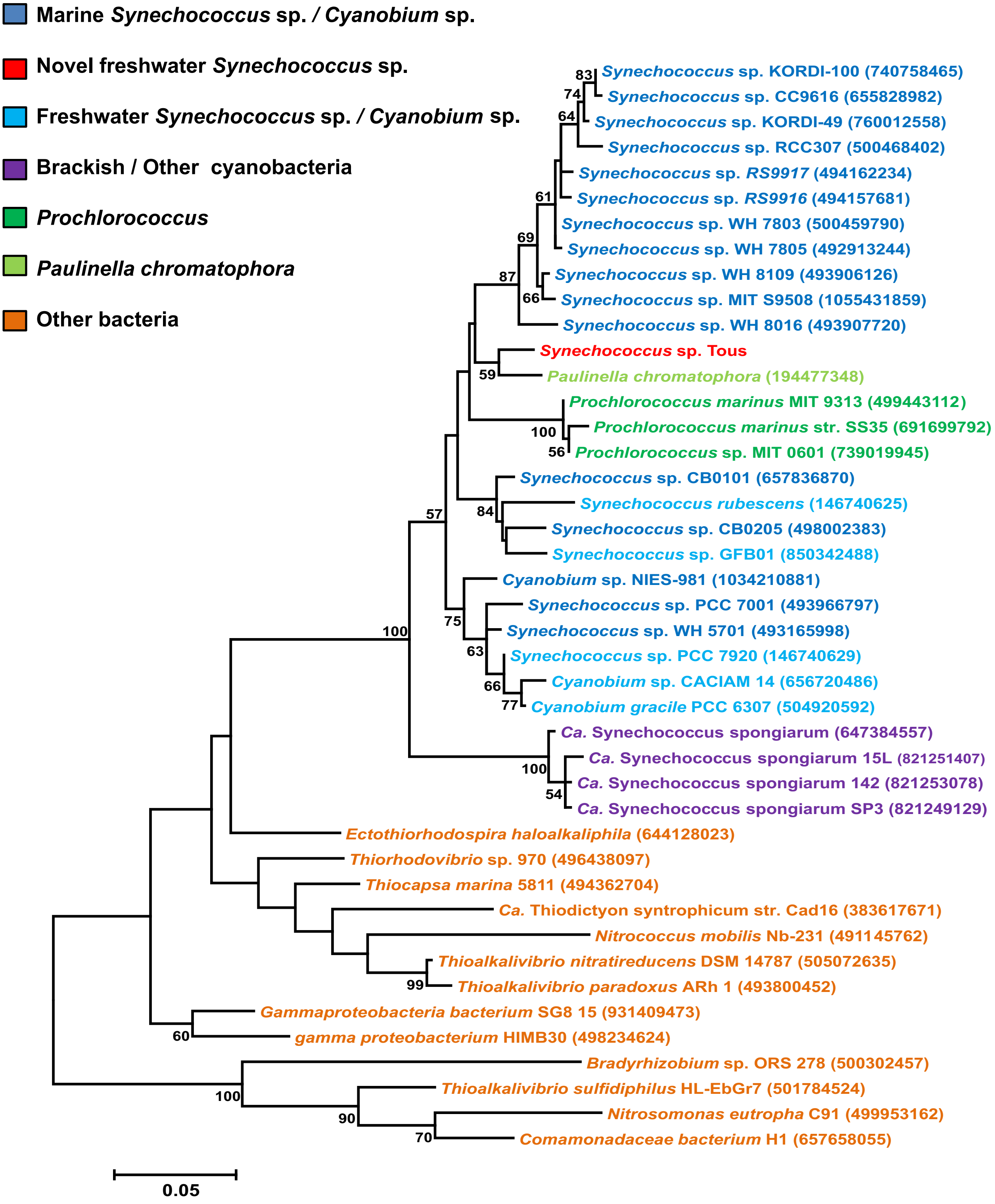

Supplementary Figure S5. RuBisCo large subunit Phylogenetic within the closest *Synechococcus, Cyanobium* and the freshwater filamentous amoeba *Paulinella chromatophora.*

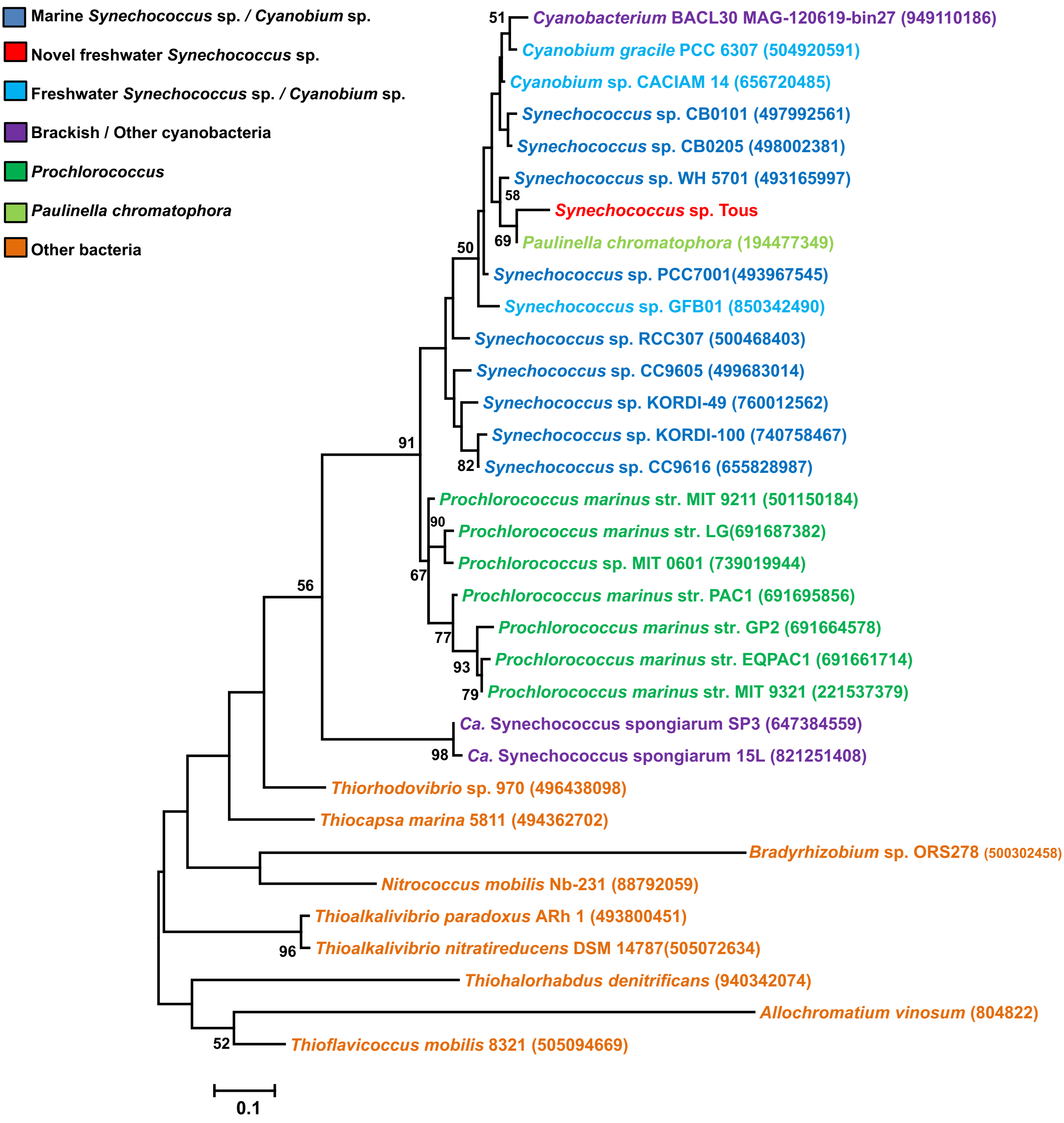

Supplementary Figure S6. RuBisCo small subunit Phylogenetic within the closest *Synechococcus, Cyanobium* and the freshwater filamentous amoeba *Paulinella chromatophora.*

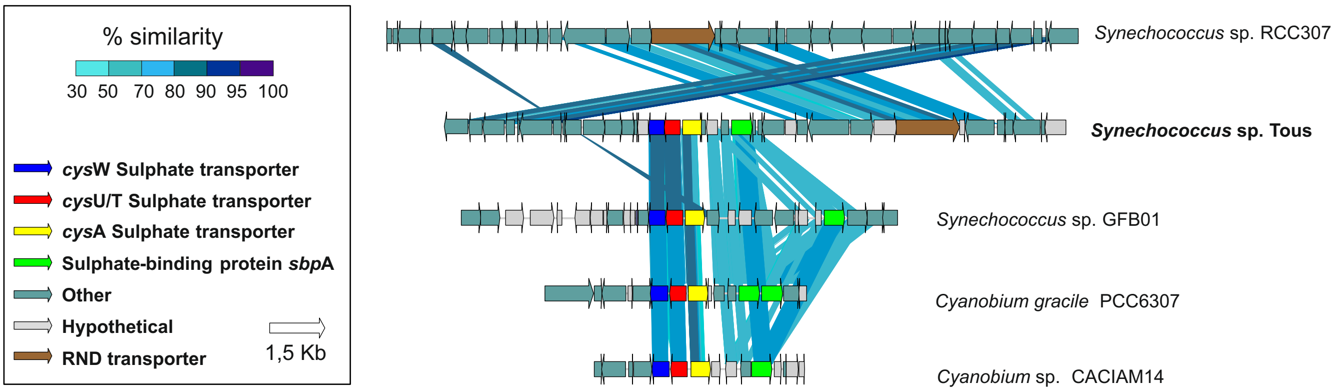

Supplementary Figure S7. Structure, synteny and conservation of *Cys* sulfate transporter gene cluster among freshwater cyanobacteria. Comparison made with TBLASTX with > 30 % of similarity hits and 150 bp of alignment lengths.

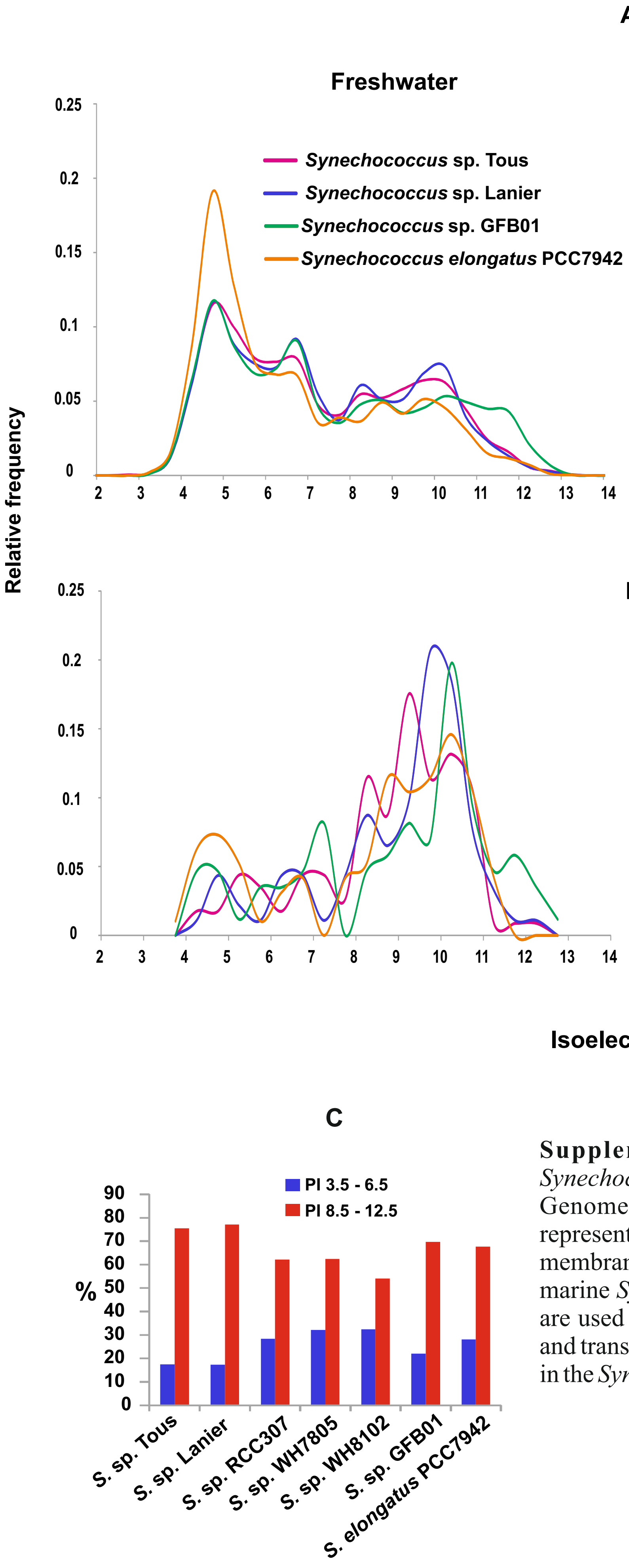

Supplementary Figure S8. A) Freshwater and marine *Synechococcus* spp. whole proteome isoelectric point profiles. Genomes are colour coded; marine *Synechococcus* spp. are represented in dashed lines. B) Isoelectric point profiles based on membrane proteins and transporters present in freshwater and marine *Synechococcus* spp. Same genomes and colour codification are used as described in 8A. C) % of different membrane proteins and transporters with isoelectric points between 3.5-6.5 and 8.5-12.5 in the *Synechococcus* spp. genomes analyzed.
